# Unlocking potential inhibitors for Bruton's tyrosine kinase through in-silico drug repurposing strategies

**DOI:** 10.1038/s41598-023-44956-0

**Published:** 2023-10-17

**Authors:** Mohammed Alrouji, Lizy Sonia Benjamin, Fahad A. Alhumaydhi, Waleed Al Abdulmonem, Saleh Salem Baeesa, Mohd Rehan, Moyad Shahwan, Anas Shamsi, Atiya Akhtar

**Affiliations:** 1 Department of Medical Laboratories, College of Applied Medical Sciences, Shaqra University, 11961 Shaqra, Saudi Arabia; 2 College of Nursing, King Khalid University (KKU), Abha, Kingdom of Saudi Arabia; 3 Department of Medical Laboratories, College of Applied Medical Sciences, Qassim University, 52571 Buraydah, Saudi Arabia; 4 Department of Pathology, College of Medicine, Qassim University, Buraydah, Saudi Arabia; 5 Division of Neurosurgery, College of Medicine, King Abdulaziz University, Jeddah, Saudi Arabia; 6 King Fahd Medical Research Center, King Abdulaziz University, 21589 Jeddah, Saudi Arabia; 7 College of Pharmacy and Health Sciences, Ajman University, Ajman, UAE; 8 Center for Medical and Bio-Allied Health Sciences Research, Ajman University, Ajman, UAE; 9 Department of Pharmacognosy, College of Pharmacy, King Khalid University (KKU), Guraiger St., 62529 Abha, Saudi Arabia

**Keywords:** Drug screening, Virtual screening

## Abstract

Bruton's tyrosine kinase (BTK) is a non-receptor protein kinase that plays a crucial role in various biological processes, including immune system function and cancer development. Therefore, inhibition of BTK has been proposed as a therapeutic strategy for various complex diseases. In this study, we aimed to identify potential inhibitors of BTK by using a drug repurposing approach. To identify potential inhibitors, we performed a molecular docking-based virtual screening using a library of repurposed drugs from DrugBank. We then used various filtrations followed by molecular dynamics (MD) simulations, principal component analysis (PCA), and Molecular Mechanics Poisson Boltzmann Surface Area (MM-PBSA) analysis to further evaluate the binding interactions and stability of the top-ranking compounds. Molecular docking-based virtual screening approach identified several repurposed drugs as potential BTK inhibitors, including Eltrombopag and Alectinib, which have already been approved for human use. All-atom MD simulations provided insights into the binding interactions and stability of the identified compounds, which will be helpful for further experimental validation and optimization. Overall, our study demonstrates that drug repurposing is a promising approach to identify potential inhibitors of BTK and highlights the importance of computational methods in drug discovery.

## Introduction

Bruton's tyrosine kinase (BTK) is a protein kinase that plays a crucial role in various biological processes, including immune system function and cancer development^1^. It is a member of the TEK family and is expressed in hematopoietic and plasma cells, which regulate signals such as MAPK (Mitogen-activated protein kinase), P13K (Phosphoinositide 3-kinase), and NF-kB (Nuclear factor kappa B) pathways. BTK also plays a key role in B-cell signaling pathways, controlling B-cell activation, maturation, proliferation, differentiation, and apoptosis^2^. The protein comprises 659 amino acids and has five domains: a PH domain, TH domain, SH2 and SH3 domain, and a catalytic domain. The PH domain plays a crucial role in regulating the interaction of proteins with phospholipids and other proteins^3^. Tyrosine 223 is the autophosphorylation site found in the SH2 and SH3 domains, while tyrosine 551 and cysteine 481 are the phosphorylation sites found in the catalytic domain that are the target of irreversible inhibitors. These structural features are essential for the development of various types of BTK inhibitors.

Tyrosine kinases (TK) have become a promising therapeutic target in the treatment of cancer due to the discovery that many cancer types may be characterized by the hyperactivation or hyperregulation of various TK^4^. In 1993, it was first reported that BTK is related to the inherited X-linked immunodeficiency disease called agammaglobulinema (XLA)^5,6^. Furthermore, its mutation causes abnormalities in transforming pre-B cells in the bone marrow into mature peripheral B cells. Moreover, its mutation causes abnormalities in transforming pre-B cells in the bone marrow into mature peripheral B cells. The immature form of B-cell receptor (BCR) that is pre-BCR transduces the signals for differentiation and growth. Therefore, any abnormalities in the function of BTK can result in hampered pre-BCR signaling and B cell development. BTK is essential for activating B cells and the counterselection of autoreactive B cells through the BCR signaling, as indicated by the various animal models^7^.

In recent years, the development of BTK inhibitors has greatly improved the treatment of inflammatory diseases and blood cancers^8^. To date, several BTK inhibitors have been approved for human use, including acalabrutinib, ibrutinib, tirabrutinib, zanubrutinib, and orelabrutinib^8^. Ibrutinib (also known as PCI-32765), discovered in 2007, is one of the first-generation BTK inhibitors approved for chronic lymphocytic leukemia (CLL). Despite high-risk gene abnormalities (IGHV mutation, TP53 mutation, and del(17p)), ibrutinib has been approved for treating various subsets of CLL, including newly diagnosed, relapsed/refractory, and elderly patients. Since 2013, ibrutinib has been approved for the treatment of mantle cell lymphoma (MCL), CLL/SLL, chronic graft-versus-host disease (GVHD), Waldenström's macroglobulinemia (WM), and marginal zone lymphoma (MZL) as monotherapy or in combination therapy^9^. The success of ibrutinib marked a turning point in treating B-cell malignancies, making it possible to treat these diseases without chemotherapy. However, it should be noted that ibrutinib can also inhibit other kinases, including the SRC-family, TEC-family, and EGFR families of receptors^10^. Second-generation BTK inhibitors, such as Zanubrutinib (BGB-3111), Calabrutinib (ACP-196), Tirabrutinib (ONO/GS-4059), and Orelabrutinib (ICP-022), are designed to have a lower impact on off-target kinases while maximizing selective BTK occupancy and effectiveness^11^. Acalabrutinib has demonstrated greater specificity compared to ibrutinib in in vitro studies. It exhibits selectivity that is 323-fold, 94-fold, 19-fold, and ninefold higher than other kinases like ITK, TXK, BMX, and TEC, respectively^12^.

Since the discovery of ibrutinib, the selectivity of BTK inhibitors has improved, but there is still room for further improvement. One of the most commonly reported side effects of ibrutinib is atrial fibrillation, which is one of the main reasons for better selectivity in BTK inhibitors^13^. It has been observed that when ibrutinib combines with other kinases, such as those in the EGFR and TEC families, it can result in adverse reactions due to its off-target effects. Therefore, it is crucial to find small molecule inhibitors of BTK that have low cytotoxicity and high efficacy to develop the treatment of BTK-associated complexities, including B-cell malignant tumors.

The discovery of new drugs is a challenging and time-consuming process, often involving screening hundreds of thousands of compounds^14,15^. Traditional experimental approaches to drug discovery, such as high-throughput screening, can be costly and may not efficiently identify the most promising drug candidates. In recent years, computational methods have emerged as powerful tools to accelerate the drug discovery process^16,17^. These methods, such as virtual screening, molecular docking, and MD simulations, can filter through large libraries of compounds and identify those most likely to bind to a target protein and possess the desired properties^18^. Also, developing new drugs from scratch targeting BTK is a complex and time-consuming process. Therefore, drug repurposing has emerged as a promising approach for identifying potential inhibitors of BTK.

In this study, we aimed to identify potential inhibitors of BTK by using a drug-repurposing approach. We performed a molecular docking-based virtual screening using a library of FDA-approved drugs. The virtual screening approach allows us to efficiently evaluate the binding interactions of large numbers of compounds with BTK. We then used all-atom molecular dynamics (MD) simulations followed by principal component analysis (PCA) and Molecular Mechanics Poisson Boltzmann Surface Area (MM-PBSA) to further evaluate the top-ranking compounds' binding interactions and stability. We have provided insights into the binding interactions and stability of the identified compounds, which will be helpful for further experimental validation and optimization. We also highlighted the importance of computational methods in drug discovery and the potential of repurposing existing drugs for new therapeutic uses.

## Materials and methods

### Virtual screening resources

Virtual screening is a computational method used in drug discovery to identify potential small molecule drug candidates^19^. Virtual screening can be used at various stages of the drug discovery process, from target identification and validation to lead optimization. It is a valuable tool for drug discovery because it allows researchers to identify potential drug candidates quickly and efficiently from a large pool of compounds and to do so in a cost-effective manner. It involves using computer algorithms to search through chemical compounds' databases and identify those likely to bind to a specific target, such as a protein or enzyme. The goal of virtual screening is to identify compounds that have the potential to be developed into drugs and to do so more efficiently and cost-effectively than traditional methods. A multistep screening, including molecular docking and PASS evaluation, followed by MD simulations for 200 ns.

An FDA-approveddrug library was downloaded from the DrugBank database^20^. The crystal structure of BTK was downloaded from the RCSB Protein Data Bank (PDB ID: 5P9J)^21^. The structure was refined by removing water molecules and cocrystallized Ibrutinib. AutoDock tools^22^ and InstaDock^23^ were used for molecular docking purposes. PyMOL^24^ and Discovery Studio Visualizer^25^ were used for visualization and analysis purposes. GROMACS 2020 beta software suite was used for the all-atom simulations for 200 ns^26^.

### Molecular docking protocol

The crystal structure of BTK was obtained from the Protein Data Bank (PDB ID: 5P9J) and used as the starting structure for the docking simulation. Any missing atoms were added, and the structure was cleaned and minimized to remove any steric clashes or unrealistic bond lengths/angles. A set of small molecule ligands were selected from the DrugBank database to be docked to the protein. These ligands were prepared in a 3D format and minimized. The protein structure and the ligands set were imported into the AutoDock tools and InstaDock docking software package and prepared for the docking. The grid maps were generated around the whole protein using the blind search space. The ligands were docked to the protein using a flexible docking protocol in a blind search space. The grid sizes for X, Y, and Z coordinates were set to 55 Å, 70 Å, and 60 Å, respectively. The centre of the grid was selected for the axes X: − 19.92, Y: − 16.01, and Z: − 5.94 with a grid spacing of 1 Å. Other parameters for the docking protocol (e.g. energy functions and scoring) were set to default, as discussed elsewhere^23,27^. The docked poses of the ligands were scored based on the energy function. The docking poses were ranked based on the binding energy, and the best-ranking complex was selected. The p*Ki*, which represents the negative decimal logarithm of the inhibition constant^28^, was determined based on the ∆*G* parameter, employing the following formula:

$$\Delta G = {\text{RT}}\left( {{\text{Ln }}Ki_{{{\text{pred}}}} } \right)$$

$$Ki_{{{\text{pred}}}} = {\text{e}}^{{(\Delta {\text{G}}/{\text{RT}})}}$$

$${\text{p}}Ki = - {\text{log}}\left( {Ki_{{{\text{pred}}}} } \right)$$

where ∆*G* is the binding affinity (kcal mol^-1^), R (gas constant) is 1.98 cal*(mol*K)^-1^, T (temperature) is 298.15 Kelvin, and *Ki*_pred_ is the predicted inhibitory constant.

Ligand efficiency (LE) is another widely used parameter for assessing favorable ligands by comparing the average binding energy per atom^29^. The calculation of LE involves the utilization of the following formula:

$${\text{LE }} = - \Delta G/{\text{N}}$$

where LE is the ligand efficiency (kcal mol^-1^ non-H atom^-1^), ∆*G* is binding affinity (kcal mol^-1^) and N is the number of non-hydrogen atoms in the ligand.

The results were analyzed by comparing the binding energy, the binding mode and the interface residues of the protein–ligand complex. The most binding-favourable complexes were chosen as hit compounds and validated in further study of all-atom MD simulations.

### MD simulations

The protein and the protein–ligand complexes were prepared from the docking study and used as the starting structure for the all-atom MD simulations. The structures were processed using the GROMACS 2020 beta utility programs, and any missing atoms and topology were added^26^. The GROMOS96 54a6 force field and the TIP3P water model were chosen for the simulation based on their suitability for simulating proteins and small molecules. Topology parameters for Eltrombopag and Alectinib were generated through PRODRG^30^. These parameters were included in the topology files. The protein–ligand complex was placed in a cubic box filled with solvent (TIP3P water molecules) using the ‘gmx solvate’ utility of GROMACS programs. The box size was adjusted to ensure that the complex was sufficiently solvated and that the minimum distance between the complex and the box edge was at least 10 Angstroms. All three systems were neutralized by adding appropriate counterions (Na^+^ and Cl^−^). The systems were energy minimized using the steepest descent algorithm using 1500 steps for 1 ns to remove any potential steric clashes. The cutoff distance for non-bonded interactions was set to 1.2 nm. All the systems were equilibrated using 1 ns simulation runs in NVT and NPT ensembles. The temperature and pressure were coupled to a target temperature of 300 K and a target pressure of 1 bar using the Berendsen algorithm with periodic boundary conditions. Particle Mesh Ewald (PME) method was utilized for long-range electrostatic interactions. The final MD simulations were run for 200 ns with a time-step of 2 fs, using the velocity Verlet algorithm for integration. After completion, the simulation trajectory was analyzed using GROMACS analysis tools to extract information about the protein–ligand complex's structure, dynamics, and interactions. The root mean square deviation (RMSD) and root mean square fluctuation (RMSF), radius of gyration (*R*g), solvent accessible surface area (SASA), and other parameters were calculated and plotted to evaluate the stability of the complexes.

### MM-PBSA analysis

MM-PBSA (Molecular Mechanics Poisson Boltzmann Surface Area) serves as a valuable tool in the process of drug discovery by examining protein–ligand interactions. To calculate the binding free energy of the docked complexes between BTK and Eltrombopag, as well as Alectinib, the MM-PBSA approach was employed. The MD simulation trajectory provided the necessary data for conducting free energy calculations within MM-PBSA. Specifically, a stable segment of the MD trajectory, encompassing 100–110 ns, was selected as a 10 ns frame for the MM-PBSA estimation. The binding free energy of the protein–ligand complexes was determined using the ‘gmx mmpbsa’ module in GROMACS.

## Result and discussion

### Molecular docking-based virtual screening

Molecular docking-based virtual screening is a computational technique used to identify potential candidate compounds for drug development from a library of compounds^18^. It involves using computer algorithms to analyze the structures of the compounds in the library and predict which ones are likely to bind to a specific target. It has been a useful tool for drug discovery as it allows researchers to quickly and efficiently identify compounds with a high likelihood of being active against a particular target. The results of the docking study indicate that approximately 3000 drugs were screened for their ability to bind to the target protein BTK. The docking scores and binding prototypes for each compound were determined, and the top five hits were selected based on their binding affinity with BTK1. These hits had a binding affinity of ≤  − 10.1 kcal/mol, as shown in Table 1. Additionally, the top 100 hits and their docking score with BTK are enlisted in Supplementary Table S1. At the same time, the top ten docked poses and their docking energies of selected five drugs against BTK are shown in Supplementary Table S2. We also calculated the binding affinities of the slected drugs in five independent runs of AutoDock Vina, each with a unique random seed (Supplementary Table S3). The results suggest that two (Eltrombopag and Alectinib) of the selected hits have significant LE values (> 0.30 kcal/mol/non-H atom) with BTK, which is a promising indication for further examination of these compounds in the drug development process^29,31^. These compounds may potentially be explored as therapeutic agents for treating diseases related to BTK, such as cancer and immune disorders. In summary, this screening has identified a set of compounds with a high binding affinity to BTK1, which may have potential as therapeutic agents.

**Table 1:** List of top five drugs selected based on the docking score and Ligand Efficiency toward BTK. Ibrutinib was taken as a reference molecule for docking study.

S. No.	Drug	Affinity (kcal/mol)	pKi	Ligand efficiency (kcal/mol/non-H atom)	Torsional energy
1	Ergotamine	− 11.1	8.14	0.26	1.5565
2	Eltrombopag	− 10.6	7.77	0.32	2.1791
3	Alectinib	− 10.6	7.77	0.30	0.9339
4	Irinotecan	− 10.2	7.48	0.24	1.8678
5	Nilotinib	− 10.1	7.41	0.26	2.1791
6	Ibrutinib	− 9.7	7.11	0.29	1.8678

### Interaction analysis

**Figure 1:**
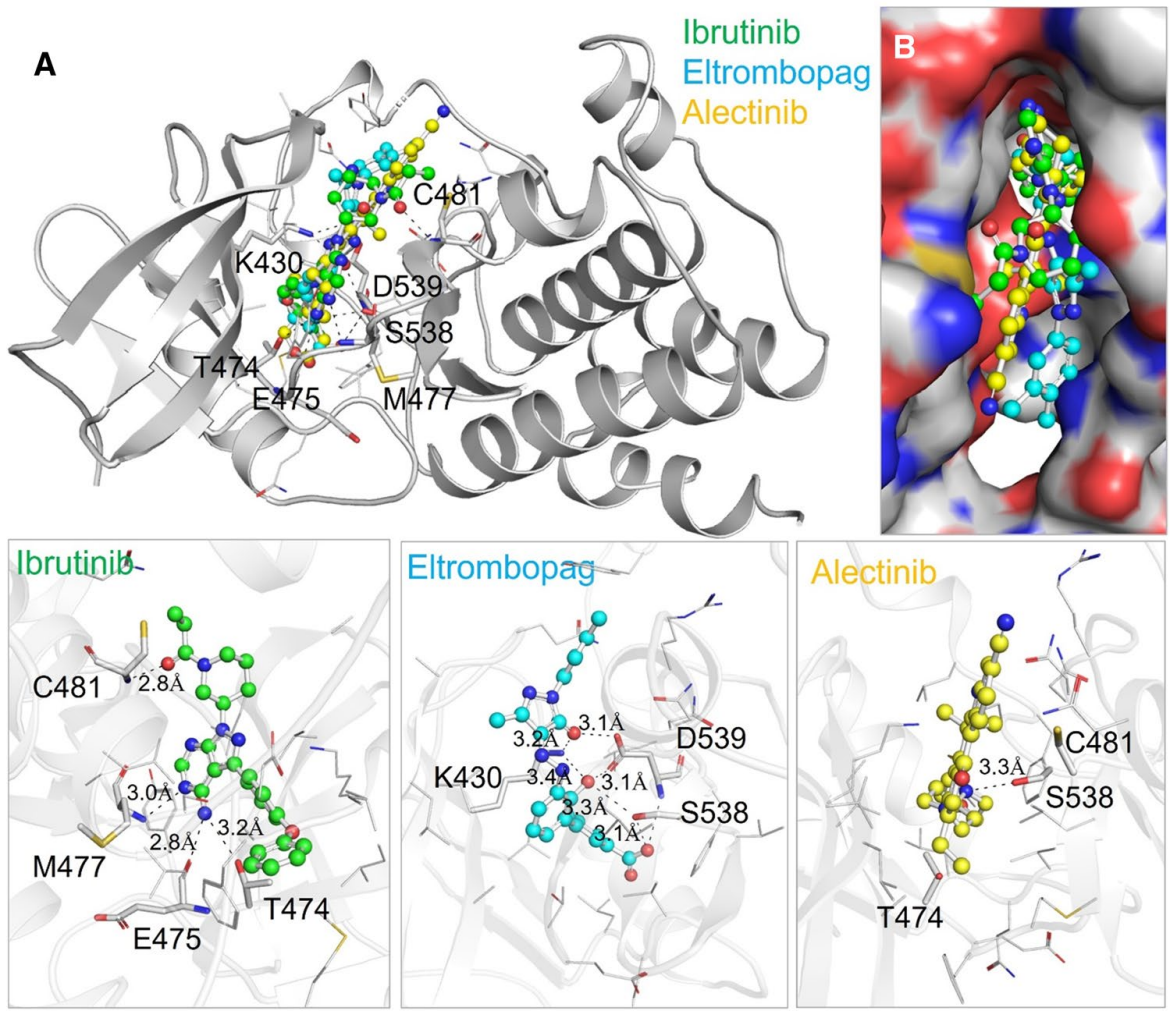
(**A**) The protein–ligand interactions in a cartoon view (**B**) Surface view of BTK binding pocket occupied by the identified drugs and the reference molecule, Ibrutinib. The interacting residues are labelled when making hydrogen bonds within 3.5 Å during the interactions. Lower panels show the magnified view of the interactions with hydrogen bonds labelled.

Interaction analysis of the selected molecules, Eltrombopag and Alectinib, were conducted to get insights into their binding mechanism with BTK. The interaction analysis revealed that both drugs interact with the ATP-binding site of BTK, specifically the Lys430 residue, as shown in Fig. 1. The figure illustrates the close interaction of Eltrombopag and Alectinib with the Lys430, Cys481, Asp539, which is the crucial region for the functional activity of BTK. Furthermore, the drugs exhibit a strong binding affinity by fitting well into the deep binding pocket of BTK, indicating good complementarity between the drugs and the target site (Fig. 1A). This strong binding affinity suggests that Eltrombopag and Alectinib possess a higher likelihood of effectively interacting with BTK compared to the reference drug Ibrutinib. Eltrombopag and Alectinib show several close interactions with the crucial residues of the BTK binding pocket, which is responsible for the functional activity of the protein. Both drugs share the same binding binding patterns as the reference molecule Ibrutinib with many common interactions. The Ibrutinib occupies a position at a distance of 2.8 Å, 3.0 Å, 2.8 Å and 3.2 Å from Cys481, Met477, Glu475 and Thr474, respectively, which is essential for the hydrogen bond formation (Fig. 1, lower left). At the same time, Eltrombopag occupies a position at distance of 3.2 Å and 3.4 Å with Lys430, 3.3 Å and 3.1 Å with Ser538, and 3.1 Å and 3.1 Å with Asp539, (Fig. 1, lower middle). In contrast, Alectinib occupies a position at a distance of 3.3 Å from Ser538, which is essential for the hydrogen bond formation (Fig. 1, lower right).

Previous studies have suggested that radicicol binds to and inhibits the ATP-binding pocket of BTK by interacting with Lys430 and Thr474 residues^21,32^. Interestingly, Eltrombopag and Alectinib, both of which bind to the same pocket, also form polar interactions with Lys430 and Thr474. These interactions are crucial for the catalytic activity of BTK. Disruption of these interactions has been shown to diminish or greatly reduce the enzyme's catalytic activity^32^. Therefore, the binding of Eltrombopag and Alectinib within the ATP-binding cavity stabilizes the interactions and can have a significant impact on the catalytic activity of BTK.

**Figure 2:**
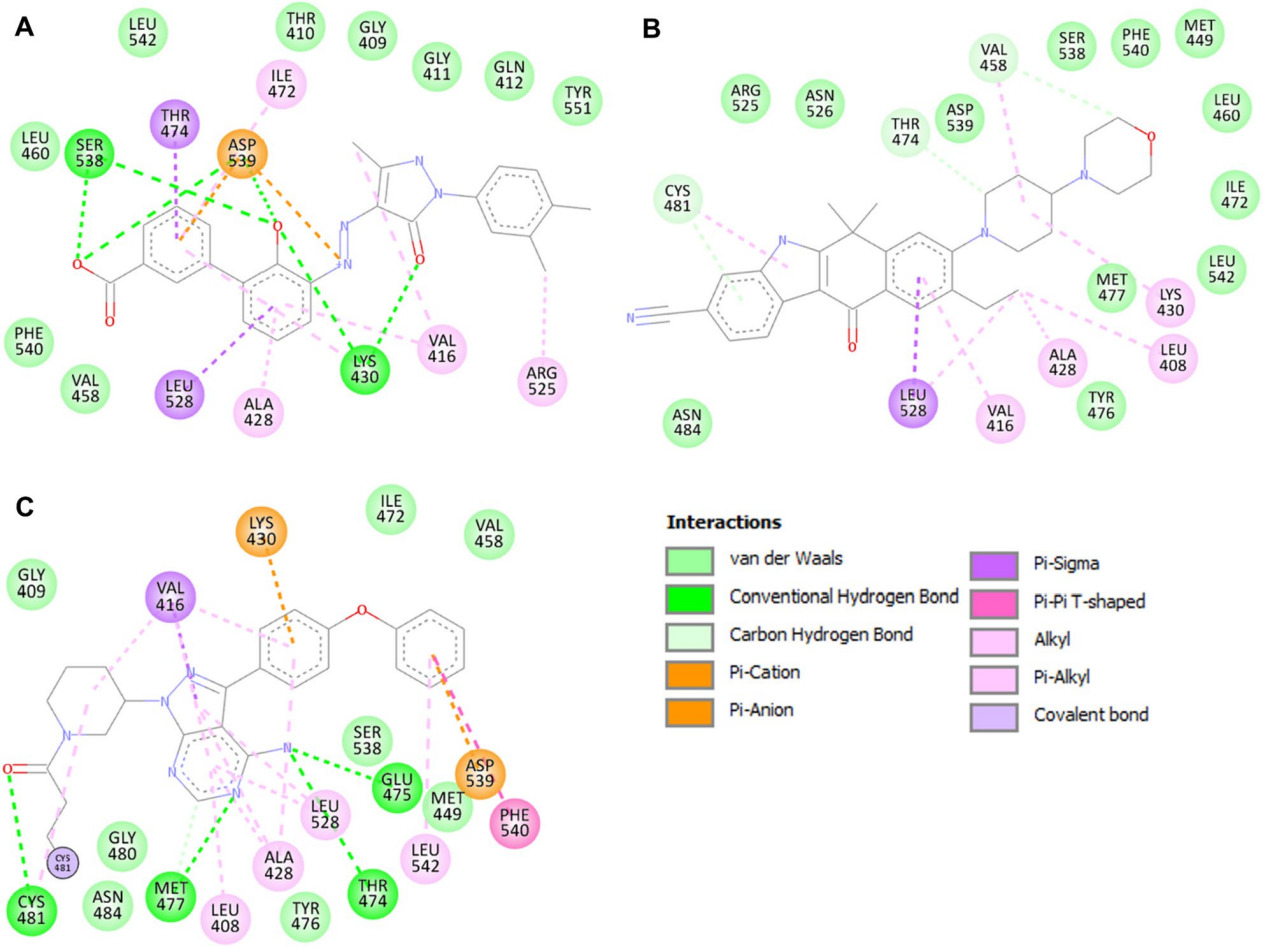
2D diagrams of BTK interactions with (**A**) Eltrombopag (**B**) Alectinib (**C**) Ibrutinib.

To further investigate the interactions of Eltrombopag and Alectinib with the binding site residues, we used Discovery Studio Visualizer. Figure 2 shows the interactions of both drugs along with Ibrutinib with the Lys430, the ATP-binding site, indicating that they could act as potential ATP competitive inhibitors of BTK. Altogether, the study suggested Eltrombopag and Alectinib can act as potential inhibitors of BTK through interaction analysis. The study found that both drugs interact with the functionally important site of BTK, and bind in a good complementarity in the deep binding pocket of BTK.

Previous studies have suggested that inhibitors bind to the ATP-binding pocket of BTK and interact with Lys430 and Thr474^33^. These interactions play a critical role in the catalytic activity of BTK, and disruption of these interactions has been shown to impair BTK's enzymatic function. The binding of Eltrombopag and Alectinib within the ATP-binding cavity stabilizes these interactions and can significantly impact BTK's catalytic activity. This suggests that Eltrombopag and Alectinib may have potential as therapeutic agents for treating diseases related to BTK, such as cancer and immune disorders. However, further studies must confirm their binding efficiency and stability with BTK.

### MD simulations

MD simulation can be used to study a wide range of phenomena in drug discovery, including the binding of small molecule drugs to protein targets^34^. These conformational changes occur in proteins upon drug binding^35^. One of the main advantages of MD simulation is that it allows researchers to study the behaviour of a system at the atomic or molecular level, providing detailed insights that are difficult to obtain using other techniques. Here, MD simulations were conducted to evaluate these compounds' binding interactions and stability. MD simulations showed that Eltrombopag and Alectinib bind to BTK with high affinity and stability. The simulations also revealed the detailed molecular interactions between the compounds and BTK, including hydrogen bonding and electrostatic interactions. The stability of the complexes was also evaluated by calculating the RMSD, RMSF, *R*g, SASA, etc., parameters of the complexes. The results showed that the complexes were stable over the simulation time. We also calculated the hydrogen bonding and PCA to evaluate the structural compactness of the complexes.

### Root mean square deviation (RMSD)

**Figure 3:**
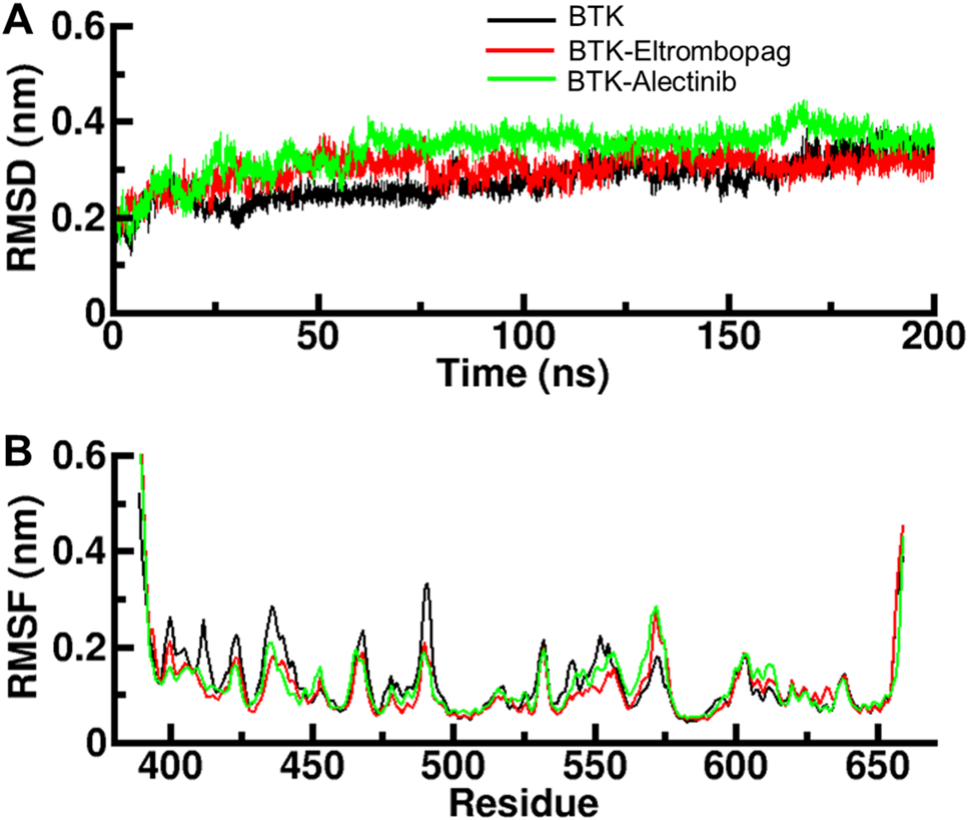
Structural dynamics of BTK upon binding with Eltrombopag and Alectinib. (**A**) Time evolution of RMSD values during the simulation. (**B**) RMSF values of each residue in BTK during the simulation.

RMSD measures the structural difference between two sets of coordinates, such as two protein–ligand complexes^36^. It is commonly used to evaluate the accuracy of protein–ligand docking predictions and to compare the binding mode of a ligand in different complexes or conformations. In the context of the protein–ligand complex, RMSD is calculated as the root mean square distance between the atoms of the protein and the ligand in the reference complex and the corresponding atoms in the predicted complex. RMSD can also be used to compare the binding mode of a ligand in different complexes or conformations, which can provide insights into the conformational changes in binding. We examined the structural dynamics of BTK before and after ligand binding. We examined RMSD to evaluate the stability of the docked complexes of BTK with Eltrombopag and Alectinib. The results showed that the binding of these ligands with BTK was equilibrated during the simulation, indicating good stability of the complexes. As shown in the RMSD graph (Fig. 3A), the BTK-Alectinib complex had a slight fluctuation before the 60 ns period but remained stable for the rest of the simulation. The BTK- Eltrombopag complex was stable with minimal fluctuation before showing random fluctuation between 140 and 160 ns. The binding of Eltrombopag to BTK exhibits minimal RMSD values at multiple regions, indicating a high degree of stability within the BTK-Eltrombopag complex throughout the 200 ns MD simulation. This observation suggests the complex remains well-equilibrated during the simulation, reinforcing its overall stability. However, during the entire simulation of 200 ns, the RMSD of all the systems remained balanced, with only a slight change seen in the BTK-Eltrombopag complex without any significant shift. These findings suggest that both the BTK-Eltrombopag and BTK-Alectinib complexes maintained structural stability during the simulation, indicating a strong interaction between the ligands and the target protein. The limited deviations in RMSD values further support the stability of the complexes, implying that the docked ligands remained in relatively stable conformations throughout the simulation period.

### Root-mean-square fluctuation

RMSF is a measure of the deviation of the coordinates of atoms in a structure from their mean positions^37^. In the context of protein–ligand simulations, RMSF is often used to assess the flexibility of the protein and ligand, specifically how much the molecule's atoms fluctuate over time in the simulated trajectory. A lower RMSF value indicates that the atoms of the protein or ligand move less, indicating that the region is more rigid, and a higher RMSF value means the region is more flexible. RMSF is usually calculated for the heavy atoms of the protein and ligand and is commonly applied on the backbone and side-chain atoms of the protein. RMSF values can help to identify residues that are likely to be involved in binding. Here, RMSF plot (Fig. 3B) was used to evaluate the residual dynamics of BTK before and after binding with Eltrombopag and Alectinib. The RMSF fluctuation was significantly reduced and stabilized upon binding with Eltrombopag, indicating a remarkably stable protein–ligand system. However, the residual vibrations upon binding with Alectinib were not uniform; in some regions, the residual vibrations were slightly increased, while in others, they were decreased, which could indicate the presence of loop regions. When comparing the RMSF values, it was observed that the BTK-Eltrombopag complex was more stable than the BTK-Alectinib complex. This suggests that Eltrombopag may have a more favorable binding interaction with BTK, leading to a more stable complex compared to Alectinib. In summary, the RMSF analysis provides valuable information regarding the flexibility and stability of the protein–ligand complexes. The reduced and stabilized RMSF values in the BTK-Eltrombopag complex indicate a highly stable interaction, while the non-uniform RMSF fluctuations in the BTK-Alectinib complex suggest varied flexibility in different regions. These findings support the notion that Eltrombopag exhibits a more favorable binding interaction with BTK, leading to a more stable complex compared to Alectinib.

### Radius of gyration

**Figure 4:**
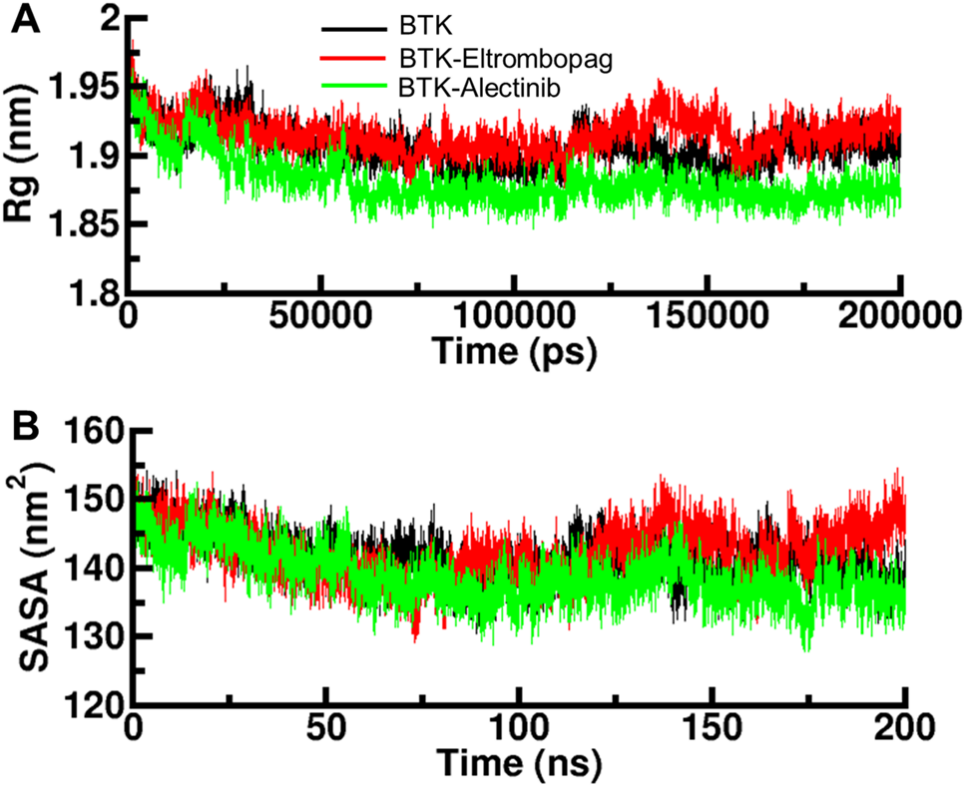
Structural compactness of BTK upon binding with Eltrombopag and Alectinib. (**A**) Time evolution of R_g_ values during the simulation. (**B**) Time evolution of SASA values during the simulation.

*R*_g_ measures the size and compactness of a macromolecular structure, such as a protein or protein–ligand complex^38^. It can be used to analyze the overall size and shape of the complex as well as the effect of the ligand binding on the structural changes of the protein. The *R*_g_ can also be used to estimate the protein's flexibility by comparing the protein's *R*_g_ values in different states (e.g., free and bound forms). A larger *R*_g_ indicates a more extended structure and more flexibility, while a smaller *R*_g_ indicates a more compact and rigid structure. To further evaluate the structural compactness of the TBK-Eltrombopag and TBK-Alectinib complexes, we used the *R*g in time-evolution settings during the simulation (Fig. 4A). The *R*_g_ plot for the TBK- Alectinib complex indicated a slight decrease in *R*_g_ value, while the *R*_g_ value of TBK-Eltrombopag was consistent with that of the apo TBK, indicating that the binding of Eltrombopag does not affect the compactness of the protein structure. The *R*_g_ analysis provided insights into the size and compactness of the protein–ligand complexes. The slight decrease in *R*_g_ observed in the BTK-Alectinib complex suggests a more compact and potentially rigid structure upon binding, while the unchanged *R*_g_ value in the BTK-Eltrombopag complex indicates that Eltrombopag binding did not induce significant structural changes. These findings contribute to our understanding of the structural dynamics and conformational stability of the BTK complexes with Eltrombopag and Alectinib.

### Solvent accessible surface area

SASA is a measure of the exposed surface area of a molecule, such as a protein or protein–ligand complex^39^. It is calculated by measuring the surface area of the molecule that is available for interaction with solvent molecules, typically using a probe with a certain radius^40^. In the context of protein–ligand simulations, SASA can be used to analyze the exposure of different parts of the protein or the ligand to the solvent and how it changes over time. This information can be used to understand how the ligand binding could affect the protein's conformation and dynamics and its biological activity. We also evaluated the SASA of the BTK-Alectinib and BTK-Eltrombopag complexes during the simulation (Fig. 4B). The results showed that no significant change in SASA was observed for the BTK-Eltrombopag and BTK-Alectinib complexes compared to the free BTK, indicating that the binding of Eltrombopag and Alectinib does not affect the solvent accessibility of the protein. However, the BTK-Eltrombopag complex in the last showed a slight disturbance in SASA value, indicating a possible change in the protein's surface exposure upon binding with Eltrombopag. Overall, the SASA analysis provided insights into the solvent accessibility and surface exposure of the protein–ligand complexes. The stable SASA values in the BTK-Alectinib complex indicated that Alectinib binding did not significantly affect the solvent accessibility of the protein. In contrast, the BTK-Eltrombopag complex displayed a slight disturbance in SASA, suggesting a possible localized change in the protein's surface exposure upon Eltrombopag binding.

### Intramolecular hydrogen bonds

**Figure 5:**
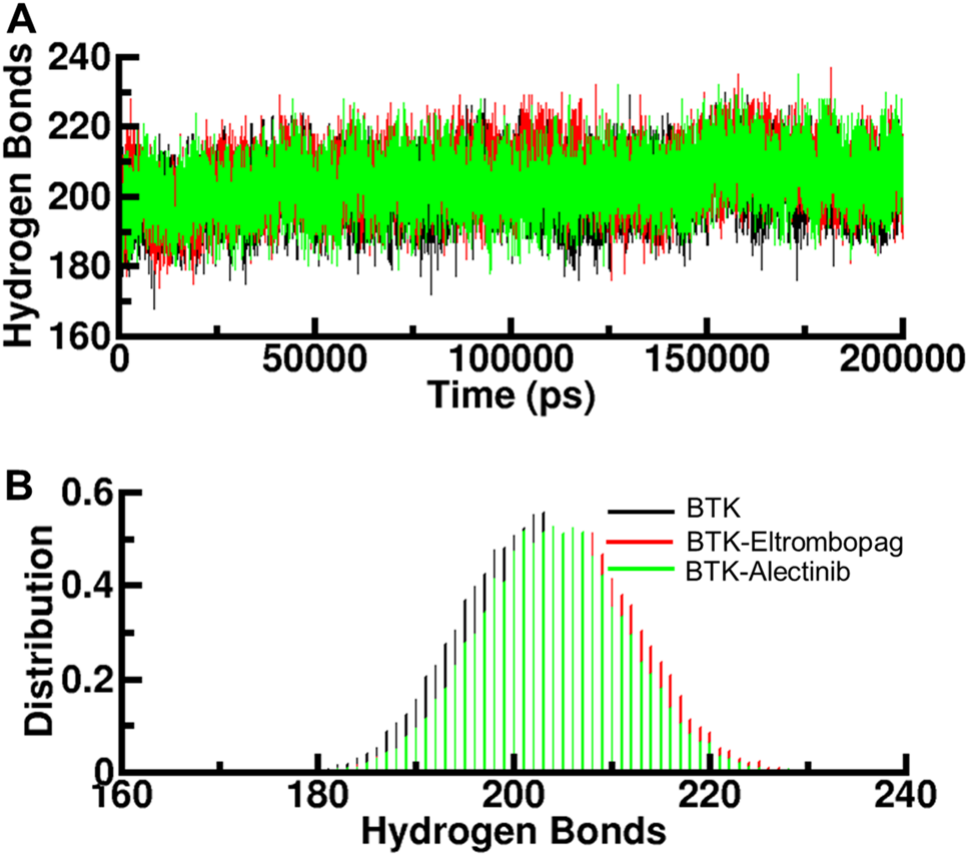
Hydrogen bond analysis. (**A**) Time evolution of intramolecular hydrogen bonds in BTK and (**B**) PDF distribution plot.

Hydrogen bonding plays a crucial role in the conformational dynamics of proteins; therefore, we analyzed the time evolution of intramolecular H-bonds to evaluate the folding dynamics of BTK in complexes with Eltrombopag and Alectinib (Fig. 5A). The plot indicated a slight increase in hydrogen bonding in the BTK complex with Eltrombopag and Alectinib. To further evaluate the consistency of the data, we calculated the probability distribution function (PDF) of the values for the three systems (Fig. 5B). The PDF plots showed good consistency. A slight change in intramolecular H-bonds was observed in the Eltrombopag and Alectinib complexes compared to the free BTK. This suggests that these compounds may affect the protein's conformation after the binding events. Overall, the analysis of intramolecular hydrogen bonds provided evidence that the binding of Eltrombopag and Alectinib to BTK influenced the protein's conformation. The increased hydrogen bonding suggests a potential stabilization effect and conformational changes induced by the ligands.

### Intermolecular hydrogen bonding

**Figure 6:**
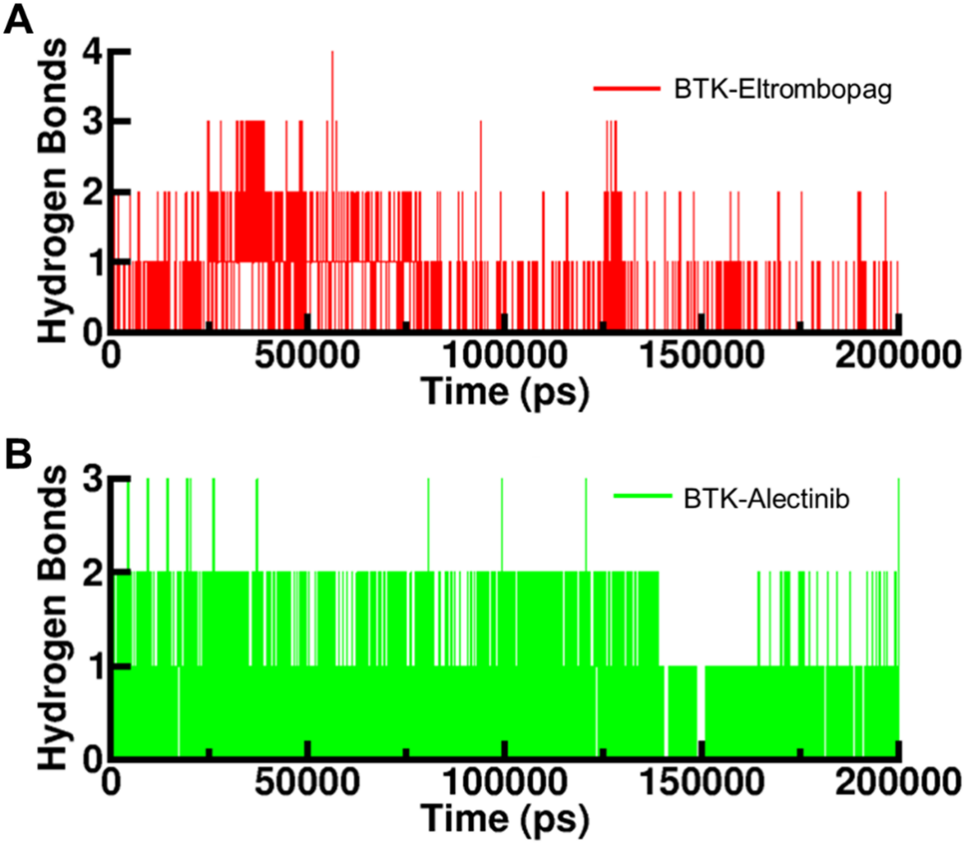
Time-evolution of intermolecular hydrogen bonds between BTK and elucidated drugs.

Hydrogen bonds play a crucial role in stabilizing protein–ligand complexes^41^. We analysed the time evolution of intermolecular hydrogen bonds to evaluate the strength of hydrogen bonding between the compounds and BTK (Fig. 6). The average number of hydrogen bonds formed in the BTK-Eltrombopag and BTK-Alectinib complex was found to be two. However, in the BTK- Eltrombopag complex, hydrogen bonding was observed to occur in phases during the entire simulation (Fig. 6A). Analysis of hydrogen bonds during the MD simulation provided insights into the binding of Eltrombopag and Alectinib within the BTK binding pocket. The results revealed that both compounds form 2–4 hydrogen bonds with higher fluctuations, indicating dynamic interactions within the pocket.

Additionally, they establish 1–2 hydrogen bonds with minimal fluctuations, suggesting more stable and consistent interactions. These findings align with the results obtained from molecular docking, further supporting the reliability and accuracy of the docking predictions. The study suggests that the hydrogen bonding in the BTK-Alectinib complex was more stable than that in the Eltrombopag complex, and the initial docking position of Eltrombopag was unchanged (Fig. 6B). The study indicated that the BTK-Eltrombopag and BTK-Alectinib complexes remained stable during the 200 ns simulation.

### Principal component analysis

**Figure 7:**
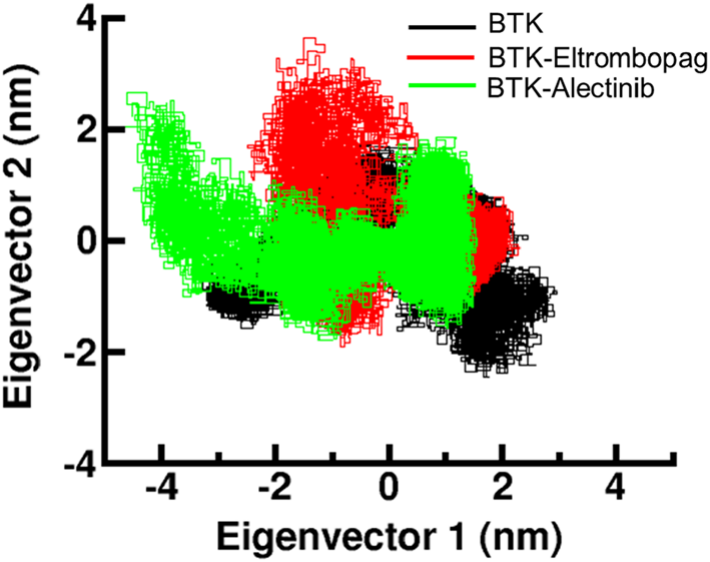
Principal component analysis of conformational projections of BTK and its complexes.

Proteins execute their specific functions through coordinated atomic motions, which can serve as a valuable parameter for comprehending protein stability. PCA is a mathematical technique widely used to analyse the dynamics of protein–ligand simulations^42^. PCA aims to identify the dominant modes of motion of a system, which can be thought of as the “principal components” of the system's motion. By analyzing these principal components, we can get insights into the structural and dynamical properties of the protein–ligand complex, such as the binding pocket, the modes of ligand binding and the ligand-induced conformational changes of the protein. It can be used to identify important structural and dynamic system features and to gain a deeper understanding of the underlying biochemistry. We used PCA to investigate the conformational changes of BTK before and after binding to Eltrombopag and Alectinib (Fig. 7). The graph shows that the conformations of BTK are projected onto two different eigenvalues based on its Cα atoms. The plot indicates that the projections of the complexes BTK-Eltrombopag and BTK-Alectinib, overlap with the conformations of the free BTK. However, it was observed that both complexes occupied slightly distinct conformational spaces, indicating that they have different structural stability. The PCA analysis provides valuable insights into the structural and dynamic behavior of the protein–ligand complexes. It helps us understand how the ligands influence the conformational landscape of BTK, revealing their impact on the stability and dynamics of the complex. These findings contribute to our understanding of the binding mechanisms and can guide further investigations into the structural and functional implications of BTK binding with Eltrombopag and Alectinib.

### Free energy landscape

**Figure 8:**
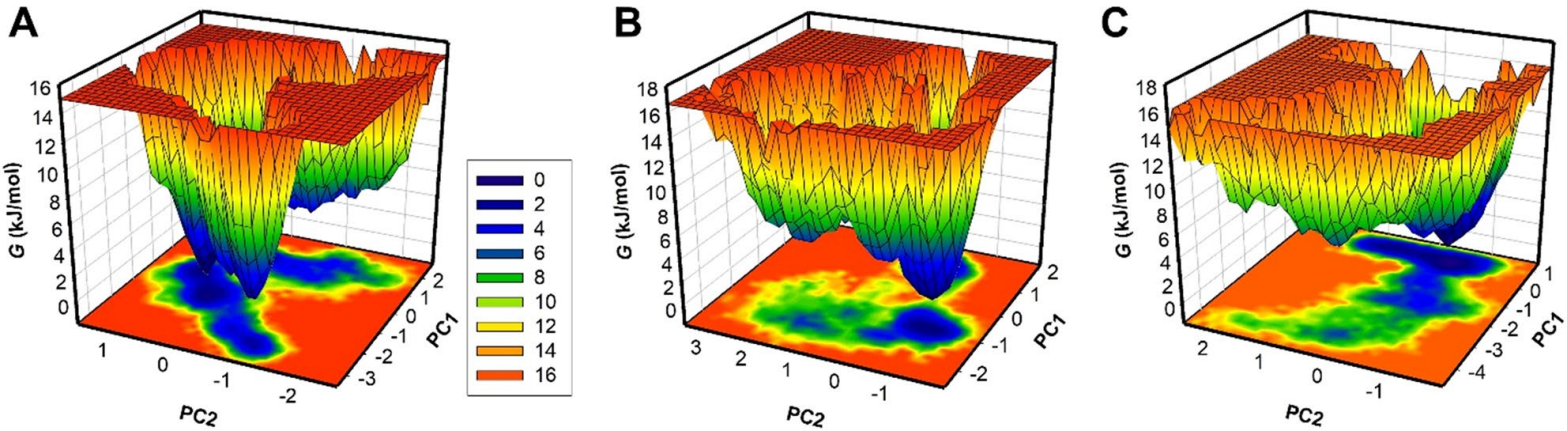
Free energy landscapes of (**A**) free BTK, (**B**) BTK-Eltrombopag and (**C**) BTK-Alectinib.

In the protein–ligand simulation, the free energy landscape (FEL) refers to the potential energy of a protein–ligand complex as a function of the positions and orientations of the protein and ligand atoms^43^. The FEL can be used to predict the most likely binding modes and affinities of a protein's ligand and identify potential binding sites on the protein. PCA is based on computing the difference in free energy between the bound and unbound states, and as such, it can give insights into the energetics of the binding process. We used FELs to analyze the energy minima and conformational landscapes of BTK and its complex systems with Eltrombopag and Alectinib. The FELs, generated using the first two PCs, were used to represent the lower energy conformations of the protein, as depicted by the deeper blue color (Fig. 8). The free BTK was observed to have multiple local minima with large basins before attaining its global minimum (Fig. 8A). The binding of Eltrombopag and Alectinib was found to slightly alter the size and position of the local and global minima of BTK, resulting in different states and multiple basins, respectively (Fig. 8B,C). The FEL plots suggested that both complexes were stable, reaching the lowest minimum conformation and not leading to abnormal unfolding. Overall, the FEL analysis provides valuable insights into the energetics and conformational landscapes of the protein–ligand complexes. It demonstrates that the binding of Eltrombopag and Alectinib altered the energy landscape of BTK, indicating stable and favorable binding interactions.

### MM-PBSA analysis

The MM-PBSA approach was employed to assess the binding free energy of BTK-Eltrombopag and BTK-Alectinib. From the stable region of the MD trajectories, a 10 ns frame was selected for the MM-PBSA estimation. The results revealed that both ligands exhibited favorable binding, as evidenced by their negative binding free energy values. Among the two, Eltrombopag displayed the lowest binding energy of − 106.36 kJ/mol, whereas Alectinib had a binding energy of − 92.82 kJ/mol. The negative binding energy was primarily driven by electrostatic interactions, non-polar solvation energy, and Van der Waals forces, while polar solvation energy made a positive contribution to the overall binding energy. These findings indicate that Eltrombopag holds greater promise as a potential inhibitor compared to Alectinib. Importantly, these conclusions align with all the simulation analyses conducted.

Taken together, the results from the MD simulations and MM-PBSA provide valuable insights into the binding interactions and stability of the identified compounds with BTK, which will be helpful for further experimental validation and optimization.

## Conclusion

BTK plays a crucial role in immune system function and cancer development, making it an attractive target for therapeutic intervention. This study aimed to identify potential inhibitors of BTK using a drug-repurposing approach. A molecular docking-based virtual screening was performed using a library of repurposed drugs from DrugBank, followed by various filtrations and MD simulations to evaluate the binding interactions and stability of the top-ranking compounds. The study results demonstrate that drug repurposing is a promising approach to identifying potential inhibitors of BTK. The molecular docking-based virtual screening approach identified several repurposed drugs as potential BTK inhibitors, including Eltrombopag and Alectinib, which have already been approved for human use. This highlights the potential for drug repurposing in the discovery of new treatments for diseases, as it allows for the re-evaluation of existing drugs for new indications. This approach can save time and resources compared to traditional drug discovery methods, as these drugs have already undergone extensive safety and efficacy testing. Furthermore, the MD simulations provided insights into the binding interactions and stability of the identified drugs, which will be helpful for further experimental validation and optimization. The simulations revealed the binding interactions between the identified molecules and BTK and allowed for the analysis of the conformational changes that occur upon binding. This information can be used to design and optimize new inhibitors with improved binding properties and stability. The findings of this study also highlight the importance of computational methods in drug discovery. The use of molecular docking and MD simulations allowed for the in silico screening and evaluation of many compounds, which would be impractical to do experimentally. These methods provide valuable insights into potential inhibitors' binding interactions and stability, which can guide further experimental studies.

### Supplementary Information

Supplementary Tables.

## Data Availability

All data generated or analyzed during this study are included in this published article.
