# Supplementary material for: Unlocking potential inhibitors for Bruton's tyrosine kinase through in-silico drug repurposing strategies
Source: Sci Rep. 2023 Oct 17;13:17684. doi: 10.1038/s41598-023-44956-0 (PMC10582150; doi:10.1038/s41598-023-44956-0)
Supplement: Supplementary file 1 — Supplementary Tables. [file 41598_2023_44956_MOESM1_ESM.docx]

*Supplementary Data*

Unlocking potential inhibitors for Bruton's tyrosine kinase throughin-silico drug repurposing strategies

Mohammed Alrouji^1^, Lizy Sonia Benjamin^2^, Fahad A Alhumaydhi^3^, Waleed Al Abdulmonem^4^, Saleh Salem Baeesa^5^, Mohd Rehan^6^, Moyad Shahwan^7,8^, Anas Shamsi^8^*, Atiya Akhtar^9^*

*^1^Department of Medical Laboratories, College of Applied Medical Sciences, Shaqra University, Shaqra 11961, Saudi Arabia. Email:* [*malrouji@su.edu.sa*](mailto:malrouji@su.edu.sa)

*^2^College of Nursing, King Khalid University (KKU), Abha, Kingdom of Saudi Arabia.*

*^3^Department of Medical Laboratories, College of Applied Medical Sciences, Qassim University, Buraydah 52571, Saudi Arabia. Email:* [*f.alhumaydhi@qu.edu.sa*](mailto:f.alhumaydhi@qu.edu.sa)

*^4^Department of Pathology, College of Medicine, Qassim University, Buraydah, Saudi Arabia. Email:* [*Dr.waleedmonem@qu.edu.sa*](mailto:Dr.waleedmonem@qu.edu.sa)

*^5^Division of Neurosurgery, College of Medicine, King Abdulaziz University, Jeddah, Saudi Arabia. Email:* [*sbaeesa@kau.edu.sa*](mailto:sbaeesa@kau.edu.sa)

*^6^King Fahd Medical Research Center, King Abdulaziz University, Jeddah, Saudi Arabia 21589. Email:* [*mrehan786@gmail.com*](mailto:mrehan786@gmail.com)

*^7^ College of Pharmacy and Health Sciences, Ajman University, UAE. Email:* [*moyad76@hotmail.com*](mailto:moyad76@hotmail.com)

*^8^*Center*for*Medical*and Bio-*Allied Health Sciences, Ajman University, UAE. Email: [anas.shamsi18@gmail.com](mailto:anas.shamsi18@gmail.com)

*^9^Department of Pharmacognosy, College of Pharmacy, King Khalid University (KKU), Guraiger St., Abha 62529, Saudi Arabia. Email:* [*atkhan@kku.edu.sa*](mailto:atkhan@kku.edu.sa)

***Corresponding Author**

**Anas Shamsi, PhD**

Center for Medical and Bio-Allied Health Sciences

Ajman University

United Arab Emirates

Email: [anas.shamsi18@gmail.com](mailto:anas.shamsi18@gmail.com)

**Akhtar Atiya**

Department of Pharmacognosy,

College of Pharmacy

King Khalid University (KKU), Saudi Arabia

**Email:** [**atkhan@kku.edu.sa**](mailto:atkhan@kku.edu.sa)

**Table S1:** List of top 100 hits and their docking score with BTK.

| **S. No.** | **Name of the Ligand** | **Binding Free Energy (kcal/mol)** | **pKi** | **Ligand Efficiency (kcal/mol/non-H atom)** | **Torsional Energy** |
| --- | --- | --- | --- | --- | --- |
|  | Ergotamine | −11.1 | 8.14 | 0.26 | 1.5565 |
|  | Eltrombopag | −10.6 | 7.77 | 0.32 | 2.1791 |
|  | Alectinib | −10.5 | 7.70 | 0.30 | 0.9339 |
|  | Irinotecan | −10.2 | 7.48 | 0.24 | 1.8678 |
|  | Nilotinib | −10.1 | 7.41 | 0.26 | 2.1791 |
|  | Atovaquone | −10.0 | 7.33 | 0.39 | 0.9339 |
|  | Prasterone_Sulfate | −9.9 | 7.26 | 0.40 | 0.9339 |
|  | Bexarotene | −9.9 | 7.26 | 0.38 | 1.2452 |
|  | Hydroxyestrone Diacetate | −9.8 | 7.19 | 0.36 | 1.2452 |
|  | Quinestrol | −9.8 | 7.19 | 0.36 | 1.2452 |
|  | Irbesartan | −9.8 | 7.19 | 0.31 | 2.1791 |
|  | Ponatinib | −9.8 | 7.19 | 0.25 | 2.1791 |
|  | Calcipotriene | −9.8 | 7.19 | 0.33 | 2.4904 |
|  | Adapalene | −9.7 | 7.11 | 0.31 | 1.5565 |
|  | Paliperidone | −9.7 | 7.11 | 0.31 | 1.5565 |
|  | Nafamostat | −9.7 | 7.11 | 0.37 | 1.8678 |
|  | Paritaprevir | −9.7 | 7.11 | 0.18 | 2.1791 |
|  | Canagliflozin | −9.7 | 7.11 | 0.32 | 2.8017 |
|  | Fentonium | −9.7 | 7.11 | 0.27 | 3.113 |
|  | Anazolene | −9.7 | 7.11 | 0.23 | 3.4243 |
|  | Daclatasvir | −9.7 | 7.11 | 0.18 | 4.0469 |
|  | Pentamycin | −9.7 | 7.11 | 0.21 | 4.6695 |
|  | Ethisterone | −9.6 | 7.04 | 0.42 | 0.3113 |
|  | Promegestone | −9.6 | 7.04 | 0.40 | 0.6226 |
|  | Conivaptan | −9.6 | 7.04 | 0.25 | 1.2452 |
|  | Risperidone | −9.6 | 7.04 | 0.32 | 1.2452 |
|  | Montelukast | −9.6 | 7.04 | 0.23 | 4.3582 |
|  | Quinbolone | −9.5 | 6.97 | 0.37 | 0.6226 |
|  | Aminoquinuride | −9.5 | 6.97 | 0.34 | 1.2452 |
|  | Casopitant | −9.5 | 6.97 | 0.22 | 1.8678 |
|  | Idarubicin | −9.5 | 6.97 | 0.26 | 2.4904 |
|  | Itraconazole | −9.5 | 6.97 | 0.19 | 3.4243 |
|  | Bisoctrizole | −9.5 | 6.97 | 0.19 | 3.7356 |
|  | Bictegravir | −9.4 | 6.89 | 0.29 | 1.2452 |
|  | Mizolastine | −9.4 | 6.89 | 0.29 | 1.2452 |
|  | Promestriene | −9.4 | 6.89 | 0.39 | 1.2452 |
|  | Fendosal | −9.4 | 6.89 | 0.32 | 1.5565 |
|  | Flibanserin | −9.4 | 6.89 | 0.34 | 1.5565 |
|  | Droperidol | −9.4 | 6.89 | 0.34 | 1.8678 |
|  | Netupitant | −9.4 | 6.89 | 0.23 | 2.1791 |
|  | Lapatinib | −9.4 | 6.89 | 0.23 | 3.4243 |
|  | Elbasvir | −9.4 | 6.89 | 0.14 | 4.0469 |
|  | Aclarubicin | −9.4 | 6.89 | 0.16 | 4.3582 |
|  | Lanreotide | −9.4 | 6.89 | 0.12 | 6.5373 |
|  | Rubitecan | −9.3 | 6.82 | 0.32 | 0.9339 |
|  | Fenoverine | −9.3 | 6.82 | 0.28 | 1.2452 |
|  | Piketoprofen | −9.3 | 6.82 | 0.36 | 1.5565 |
|  | Buclizine | −9.3 | 6.82 | 0.30 | 1.8678 |
|  | Midostaurin | −9.3 | 6.82 | 0.22 | 1.8678 |
|  | Fendiline | −9.3 | 6.82 | 0.39 | 2.1791 |
|  | Rifabutin | −9.3 | 6.82 | 0.15 | 2.4904 |
|  | Calcitriol | −9.3 | 6.82 | 0.31 | 2.8017 |
|  | Fluoxymesterone | −9.2 | 6.75 | 0.38 | 0.6226 |
|  | Azelastine | −9.2 | 6.75 | 0.34 | 0.9339 |
|  | Nandrolone Cyclotate | −9.2 | 6.75 | 0.30 | 0.9339 |
|  | Meprednisone | −9.2 | 6.75 | 0.34 | 1.2452 |
|  | Indoramin | −9.2 | 6.75 | 0.35 | 1.5565 |
|  | Scarlet_Red | −9.2 | 6.75 | 0.32 | 1.5565 |
|  | Dihydrotachysterol | −9.2 | 6.75 | 0.32 | 1.8678 |
|  | Estradiol Dipropionate | −9.2 | 6.75 | 0.33 | 1.8678 |
|  | Brigatinib | −9.2 | 6.75 | 0.23 | 2.4904 |
|  | Panobinostat | −9.2 | 6.75 | 0.35 | 2.4904 |
|  | Enasidenib | −9.2 | 6.75 | 0.28 | 2.8017 |
|  | Evans Blue | −9.2 | 6.75 | 0.16 | 5.2921 |
|  | Picloxydine | −9.1 | 6.67 | 0.28 | 0.6226 |
|  | Estradiol Acetate | −9.1 | 6.67 | 0.40 | 0.9339 |
|  | Ethinylestradiol | −9.1 | 6.67 | 0.41 | 0.9339 |
|  | Pimobendan | −9.1 | 6.67 | 0.36 | 0.9339 |
|  | Nicocodine | −9.1 | 6.67 | 0.30 | 1.2452 |
|  | Rilpivirine | −9.1 | 6.67 | 0.32 | 1.5565 |
|  | Belotecan | −9.1 | 6.67 | 0.28 | 1.8678 |
|  | Bisantrene | −9.1 | 6.67 | 0.30 | 1.8678 |
|  | Estradiol Valerate | −9.1 | 6.67 | 0.35 | 1.8678 |
|  | Fludrocortisone Acetate | −9.1 | 6.67 | 0.30 | 1.8678 |
|  | Cinacalcet | −9.1 | 6.67 | 0.35 | 2.1791 |
|  | Betamethasone Phosphate | −9.1 | 6.67 | 0.28 | 2.4904 |
|  | Carindacillin | −9.1 | 6.67 | 0.26 | 2.4904 |
|  | Netarsudil | −9.1 | 6.67 | 0.27 | 2.8017 |
|  | Olodaterol | −9.1 | 6.67 | 0.32 | 2.8017 |
|  | Benzquercin | −9.1 | 6.67 | 0.16 | 4.9808 |
|  | Plicamycin | −9.1 | 6.67 | 0.12 | 8.0938 |
|  | Maprotiline | −9.0 | 6.60 | 0.43 | 1.2452 |
|  | Dabrafenib | −9.0 | 6.60 | 0.26 | 2.1791 |
|  | Simeprevir | −9.0 | 6.60 | 0.17 | 2.4904 |
|  | Fidaxomicin | −9.0 | 6.60 | 0.13 | 6.8486 |
|  | Fluocinolone Acetonide | −8.9 | 6.53 | 0.28 | 1.2452 |
|  | Hetacillin | −8.9 | 6.53 | 0.33 | 1.2452 |
|  | Isotretinoin | −8.9 | 6.53 | 0.40 | 1.8678 |
|  | Ifenprodil | −8.9 | 6.53 | 0.37 | 2.1791 |
|  | Lasofoxifene | −8.9 | 6.53 | 0.29 | 2.1791 |
|  | Calcifediol | −8.9 | 6.53 | 0.31 | 2.4904 |
|  | Ipragliflozin | −8.9 | 6.53 | 0.32 | 2.4904 |
|  | Daunorubicin | −8.9 | 6.53 | 0.23 | 2.8017 |
|  | Daunorubicinol | −8.9 | 6.53 | 0.23 | 3.113 |
|  | Desmopressin | −8.9 | 6.53 | 0.12 | 6.226 |
|  | Finasteride | −8.8 | 6.45 | 0.33 | 0.6226 |
|  | Phenolphthalein | −8.8 | 6.45 | 0.37 | 1.2452 |
|  | Axitinib | −8.8 | 6.45 | 0.31 | 1.5565 |
|  | Bendroflumethiazide | −8.8 | 6.45 | 0.33 | 1.5565 |
|  | Etravirine | −8.8 | 6.45 | 0.31 | 1.5565 |

**Table S2:** The top ten docked poses and their docking energies of selected drugs against BTK.

| **S. No.** | **Drug** | **Docked**  **conformation** | **Affinity (kcal/mol)** | **RMS Distance from best mode** | |
| --- | --- | --- | --- | --- | --- |
|  |  |  |  | **Lower bound** | **Upper bound** |
|  | Ergotamine |  | −11.1 | 0.000 | 0.000 |
|  |  |  | −11.0 | 1.999 | 9.784 |
|  |  |  | −11.0 | 3.185 | 11.109 |
|  |  |  | −10.8 | 20.275 | 23.814 |
|  |  |  | −10.8 | 17.265 | 20.623 |
|  |  |  | −10.7 | 2.222 | 9.294 |
|  |  |  | −10.4 | 2.909 | 10.632 |
|  |  |  | −10.4 | 2.689 | 10.353 |
|  |  |  | −10.3 | 21.040 | 25.615 |
|  |  |  | −10.1 | 20.563 | 23.648 |
|  | Eltrombopag |  | −10.6 | 0.000 | 0.000 |
|  |  |  | −10.3 | 4.137 | 7.821 |
|  |  |  | −10.0 | 2.664 | 10.427 |
|  |  |  | −9.9 | 2.226 | 10.563 |
|  |  |  | −9.7 | 1.382 | 2.115 |
|  |  |  | −9.7 | 18.097 | 20.906 |
|  |  |  | −9.3 | 2.972 | 10.235 |
|  |  |  | −9.2 | 13.337 | 19.126 |
|  |  |  | −9.1 | 4.986 | 13.136 |
|  |  |  | −9.0 | 19.003 | 22.303 |
|  | Alectinib |  | −10.6 | 0.000 | 0.000 |
|  |  |  | −10.5 | 2.385 | 4.378 |
|  |  |  | −10.4 | 4.246 | 12.954 |
|  |  |  | −10.1 | 2.017 | 2.482 |
|  |  |  | −9.7 | 3.114 | 6.280 |
|  |  |  | −9.4 | 4.395 | 12.235 |
|  |  |  | −9.3 | 2.323 | 4.804 |
|  |  |  | −9.2 | 3.770 | 12.340 |
|  |  |  | −8.9 | 2.526 | 11.031 |
|  |  |  | −8.6 | 4.372 | 11.120 |
|  | Irinotecan |  | −10.2 | 0.000 | 0.000 |
|  |  |  | −10.1 | 1.439 | 2.112 |
|  |  |  | −9.5 | 25.434 | 28.025 |
|  |  |  | −9.5 | 3.675 | 12.327 |
|  |  |  | −9.5 | 3.632 | 13.262 |
|  |  |  | −9.4 | 2.958 | 5.320 |
|  |  |  | −9.4 | 2.158 | 4.039 |
|  |  |  | −9.3 | 2.491 | 4.577 |
|  |  |  | −9.3 | 4.964 | 11.551 |
|  |  |  | −9.2 | 4.483 | 6.603 |
|  | Nilotinib |  | −10.1 | 0.000 | 0.000 |
|  |  |  | −10.1 | 5.743 | 9.694 |
|  |  |  | −9.9 | 4.733 | 7.889 |
|  |  |  | −9.9 | 6.056 | 10.915 |
|  |  |  | −9.8 | 6.536 | 12.735 |
|  |  |  | −9.8 | 6.044 | 9.125 |
|  |  |  | −9.7 | 3.866 | 7.404 |
|  |  |  | −9.7 | 5.860 | 9.423 |
|  |  |  | −9.7 | 5.632 | 10.761 |
|  |  |  | −9.5 | 20.953 | 23.955 |
|  | Ibrutinib |  | −9.7 | 0.000 | 0.000 |
|  |  |  | −9.6 | 1.170 | 2.474 |
|  |  |  | −9.6 | 3.959 | 7.309 |
|  |  |  | −9.4 | 3.758 | 8.121 |
|  |  |  | −9.3 | 2.480 | 4.569 |
|  |  |  | −9.0 | 2.940 | 3.537 |
|  |  |  | −9.0 | 3.249 | 4.275 |
|  |  |  | −9.0 | 2.860 | 9.620 |
|  |  |  | −8.8 | 3.179 | 9.726 |
|  |  |  | −8.2 | 25.354 | 28.261 |

**Table S3****:** The binding affinities of the chosen compounds were assessed in five independent runs of AutoDock Vina, each with a unique random seed. The replicates of AutoDock Vina runs are denoted as R1, R2, R3, ..., R5.

| **S. No.** | **Drug** | **Affinity (kcal/mol)** | | | | | |
| --- | --- | --- | --- | --- | --- | --- | --- |
|  |  | ***R1*** | ***R2*** | ***R3*** | ***R4*** | ***R5*** | ***Mean*** |
|  | Ergotamine | −11.1 | −10.6 | −10.6 | −10.6 | −10.6 | −10.7 |
|  | Eltrombopag | −10.6 | −10.6 | −10.6 | −10.6 | −10.5 | −10.6 |
|  | Alectinib | −10.6 | −10.4 | −10.3 | −10.4 | −10.5 | −10.4 |
|  | Irinotecan | −10.2 | −10.2 | −10.2 | −10.2 | −10.2 | −10.2 |
|  | Nilotinib | −10.1 | −9.9 | −9.7 | −9.9 | −9.9 | −9.9 |
|  | Ibrutinib | −9.7 | −9.8 | −8.9 | −9.9 | −9.9 | −9.5 |
